# Hair Transplantation: Preventing Post-operative Oedema

**DOI:** 10.4103/0974-2077.69018

**Published:** 2010

**Authors:** Abbasi Gholamali, Pojhan Sepideh, Emami Susan

**Affiliations:** *20 Pezeshkan Building, 1315 Valiasr St. Vanak Sq., Tehran, Iran*

**Keywords:** Hair transplantation, post-operative oedema, triamcinolone

## Abstract

Swelling or oedema of forehead or eyelids is a common consequence of hair transplantation surgery. However, this results in increased morbidity and absence from work due to unaesthetic appearance. To study various physical and therapeutic modalities to reduce or completely prevent the occurrence of such oedema. Three hundred forty hair transplant patients were recruited in the study and were categorized into 8 groups depending upon the intervention employed. There were 32 dropouts in the study due to various reasons. Patients who were administered steroid with tumescent solution had the highest number of patients without oedema, with only 3 out of 117 patients developing oedema. Physical measures like position of head during sleeping, application of occlusion bands or ice packs did not show satisfactory results. Addition of triamcinolone to tumescent anaesthetic solution is a very effective technique of preventing post-operative swelling.

## INTRODUCTION

Swelling or oedema of forehead or eyelids is a common consequence of hair transplantation surgery, especially 2–6 days after the operation. In some cases, this oedema is so severe that patient cannot open his/her eyes. In rare cases, it is accompanied with ecchymosis of the eyelids (black eyes). This may cause both morbidity and a delay in returning to normal life and work. Many methods have been recommended to reduce the oedema,[1–3] which include both physical methods and administration of steroids.

Physical methods include use of firm head band, maintaining semi-lay down position postoperatively,[1] applying adhesive tapes below hair line and usage of ice packs or bags of frozen peas. Usage of steroid may be by oral route, intramuscular injection and/or adding steroid to the local anaesthetic. However, none of these methods have been found fully satisfactory. This study is an attempt to compare the efficacy of various techniques described in literature used to reduce oedema.

## MATERIALS AND METHODS

This was a prospective study involving 340 patients who underwent hair transplantation (follicular unit transplantation) between May 2001 and August 2003. Punch was not used in any of the cases. All patients had dressing for 24 hours and they all took shower after the removal of the dressing. Regardless of age, sex, baldness or hair loss, the same method was used. All patients took prophylactic systemic antibiotics from one day before the operation till 9 days after the operation.

For objective assessment of the results, grading of post-operative oedema was done as follows:

Grade 0: No oedema

Grade I: Upper forehead oedema

Grade II: Upper and lower forehead oedema

Grade III: Periorbital oedema

Grade IV: Black eyes

All patients were checked for oedema every day until 6 days after surgery. In cases where patients’ follow-up for staging was not satisfactory, he or she was removed from this study. Based on the technique employed to reduce the post-operative oedema, patients were categorized into 7 groups [Table 1].

**Table 1:** Categorization of patients as per different intervention techniques

Group I	Day prior to operation: Prednisolone 30 mg
	On the operative day: Prednisolone 25 mg
	1^st^ post-op day: Prednisolone 20 mg
	2^nd^ post-op day: Prednisolone 15 mg
	3^rd^ post-op day: Prednisolone10 mg
	4^th^ post-op day: Prednisolone 5 mg
Group II	Inj. Methylprednisolone 40 mg given intramuscularly one day before operation
Group III	Local anaesthetic solution consisted of Triamcinolone 40 mg + Xylocaine 2% was used as a local anaesthetic in recipient area
Group IV	Local tumescent anaesthetic solution consisted of Triamcinolone 40 mg + xylocaine 2% + Epinephrine 1:1000, 1 ml + Normal saline 100 ml
Group V	Patients were advised to sleep at the 45° angle after the operation
Group VI	Patients were asked to sleep supine as much as possible
Group VII	Adhesive tape or head band was applied below the hair line
Group VIII	Ice packs or bags of frozen peas were used every 20 mins for a duration of 15 mins till 24 hours after operation

## RESULTS

A total of 340 patients (males 291, females 49) were recruited in the study of whom, 32 were deleted from the study due to poor follow-up and gastric intolerance to oral corticosteroids. Age of patients ranged from 26 to 62 years (mean age 43.5 ± 7.1). Table 2 summarizes the findings in the different groups.

**Table 2:** Prevalence and severity of oedema in different groups

Oedema stage prevention methods	Grading of post-operative oedema					Comments
	No oedema (%)	I (%)	II (%)	III (%)	IV (%)	
Group I Oral steroid N = 49	20 (47.6)	15 (35.7)	7 (16.7)	– –	– –	7 dropouts from study due to digestive problems
Group II Intramuscular steroid N = 35	23 (65.7)	8 (22.9)	4 (11.4)	– –	– –	8 dropouts due to poor follow-up
Group III Steroid and Xylocaine N = 20	14 (70)	4 (20)	2 (10)	– –	– –	– –
Group IV (Triamcinolone acetonide + xylocaine 2% + Epinephrine1/1000 + normal saline N = 126	114 (97.4)	2 (1.7)	1 (0.8)	– –	– –	9 dropouts due to poor follow-up
Group V Semi-laid down method N = 57	– –	– –	49 (86)	7 (12.3)	1 (1.8)	– –
Group VI Vertical position N = 15	– –	– –	1 (14.3)	5 (71.4)	1 (14.3)	8 dropouts due to poor follow-up
Group VII Using head-band N = 25	– –	– –	21 (84)	3 (12)	1 (4)	– –
Group VIII Using ice-bag N = 37	– –	– –	32 (86.5)	5 (13.5)	– –	– –

As can be seen from the Table 1, only groups who were administered steroids had oedema-free patients. Patients who were administered steroids had less oedema than patients who followed physical methods. Further, patients who were administered steroid with tumescent solution had the highest number of patients without oedema, with only 3 out of 117 patients developing oedema.

## DISCUSSION

Several methods have been suggested to decrease the intensity and prevalence of post-hair transplantation oedema of forehead and periorbital oedema. Our study showed that physical preventive methods are not effective to control oedema in a satisfactory way. All 126 patients who had used only physical methods had some degree of post-operative oedema. Of the physical methods, the method using vertical position of the head postoperatively was the least effective. However, the number of patients in the group was small, with only 7 patients in the study.

The method found most effective was using a tumescent mixture of 100cc normal saline + xylocaine 2% + 1cc of epinephrine 1/1000 + 40 mg of Triamcinolone in the recipient area. This method gave excellent results as 97.4% of cases who received this preventive method showed no signs of oedema, and in the rest of the cases oedema was mild.

Among the other methods studied, administration of intramuscular steroid and steroid together with Xylocaine also gave good results; however, oral steroid administration did not give satisfactory results. At the same time, the gastric side effects caused by oral steroid were also another restrictive factor.

Different methods of oedema prevention have been recommended in other cranio-facial surgeries. In 1989, Griffin recommended using steroids in rhinoplasty surgeries.[4] In a two-end blind study, Hofmann showed that steroid would lead to a significant decrease in oedema and post-rhinoplasty inflammation,[5] although there have been some debate on the actual dose, effect and prescription of this drug.[6] Norwood believed that using steroid leads to a significant decrease in the prevalence and intensity of post-operative oedema.[7] A mixture of steroid and tumescent solution was first recommended for the cranio-facial surgeries by Neil Dwyer.[8] He studied 20 cases of remodeling craniofacial surgery and found that, this formula can particularly decrease the risk of postoperative perioribital oedema.[8] Our study reinforces the findings of this study.

In summary, it can be said that using a mixture of triamcinolone and tumescent solution in recipient area in hair transplantation patients has been significantly effective in prevention of post-operative oedema. The method is also simple and safe.
